# Validation of the DECA criteria for allergic conjunctivitis severity and control

**DOI:** 10.1186/s13601-020-00349-4

**Published:** 2020-10-23

**Authors:** M. Cesárea Sánchez-Hernández, Ana M. Navarro, Carlos Colás, Alfonso del Cuvillo, Joaquín Sastre, Joaquim Mullol, Antonio Valero

**Affiliations:** 1 Department of Allergy, Hospital Universitario Virgen Macarena, Seville, Spain; 2 Department of Allergy, Hospital El Tomillar, Dos Hermanas, Seville Spain; 3 Doctor Relimpio 6, 3B, 41003 Seville, Spain; 4 Department of Allergy, Hospital Clínico-Instituto de Investigación Sanitaria de Aragón, Zaragoza, Spain; 5 Rhinology & Asthma Unit; Department of Otorhinolaryngology, Hospital Universitario de Jerez, Cádiz, Spain; 6 Department of Allergy, Fundación Jiménez Díaz, Madrid, Spain; 7 Department of Medicine, Universidad Autónoma de Madrid, Madrid, Spain; 8 CIBERES, Instituto de Salud Carlos III, Madrid, Spain; 9 Rhinology Unit & Smell Clinic, Department of Otorhinolaryngology, Hospital Clinic, Institut d’Investigacions Biomèdiques August Pi i Sunyer (IDIBAPS), Universitat de Barcelona, Barcelona, Spain; 10 Department of Pneumology and Allergy, Hospital Clínic, Institut d’Investigacions Biomèdiques August Pi i Sunyer (IDIBAPS), Barcelona, Spain

**Keywords:** Allergic conjunctivitis, Allergic rhinoconjunctivitis, Conjunctivitis classification, Control, Ocular allergy

## Abstract

**Background:**

Allergic conjunctivitis (AC) is usually associated to allergic rhinitis (AR), but the severity and control of ocular symptoms should be assessed independently to improve diagnosis and treatment. The criteria from the Spanish consensus document on allergic conjunctivitis (DECA) aimed to be used as a patient-reported instrument for AC management. Here we validate these criteria for classifying AC severity and defining its control following COSMIN guidelines recommendations.

**Methods:**

Patients with moderate or severe AR [reflective total nasal symptom score (rTNSS) score ≥ 8] and concomitant AC were recruited from hospitals in Spain. Patients were classified according to the severity of ocular symptoms as mild, moderate, or severe, and classified with respect to control as controlled and non-controlled, using the DECA criteria. To validate these criteria, comparisons with the validated modified allergic rhinitis and its impact on asthma (mARIA), reflective total ocular symptom score (rTOSS), rhinitis control assessment test (RCAT), ESPRINT-15 questionnaires, a conjunctival hyperemia scale and a visual analogue scale (VAS) for ocular symptoms were performed.

**Results:**

A total of 128 patients participated in the validation. Mean age was 34.4 ± 12.1 years; 72.7% were women. The DECA criteria showed a good discriminant validity, reflecting a high capacity to differentiate between mild, moderate, and severe patients, and controlled from uncontrolled patients. A strong association between AC and AR was reflected in the comparison between the DECA and the mARIA criteria (p < 0.0001). The DECA criteria for severity and control presented satisfactory properties for longitudinal validity and responsiveness.

**Conclusions:**

Validation of the DECA criteria for severity and control of AC suggested that it can be useful in the evaluation of eye symptoms and follow-up of therapies.

## Introduction

Allergic conjunctivitis (AC) is an immunological hypersensitivity disorder of the ocular conjunctiva, predominantly mediated by an IgE mechanism [1]. AC occurs concomitantly with allergic rhinitis (AR) and other allergic disorders in most patients, but the ocular symptoms can be present without nasal involvement in 2–7% of AC patients [2, 3]. AC is a highly prevalent disease, affecting up to 40% of the adult population [1]. The most frequent symptoms are pruritus, tearing, and conjunctival hyperemia. These symptoms can cause a significant impact on quality of life (QoL), affecting sleep and resulting in emotional problems and impairment of activities of daily living or social functions, such as work productivity or performance at school [4, 5]. AC is often underdiagnosed and undertreated, as only a small proportion of patients (~ 10%) with AC symptoms seek medical advice [6]. Failure to recognize and treat ocular symptoms associated with AR can increase the burden of the disease substantially for allergy patients.

To better understand disease progression and to help assessing the effectiveness of treatment pathways, in the last two decades the concepts of ‘disease severity’ and ‘disease control’ have been established for chronic allergic diseases [7]. Thus, several patient-reported outcome (PRO) instruments have been developed to measure severity and control of AR [8, 9]. A successful and widely discussed initiative was the allergic rhinitis and its impact on asthma (ARIA) approach, which classified patients according to severity and, more recently, also to control [8, 10, 11]. The original ARIA document classified AR severity based on the impact of AR on 4 items: sleep, activities/leisure/sports, work productivity/school performance and bothersome symptoms [8]. Based on the original ARIA criteria, a severity classification for AC patients based on ocular symptoms was also proposed [12].

**Fig. 1:**
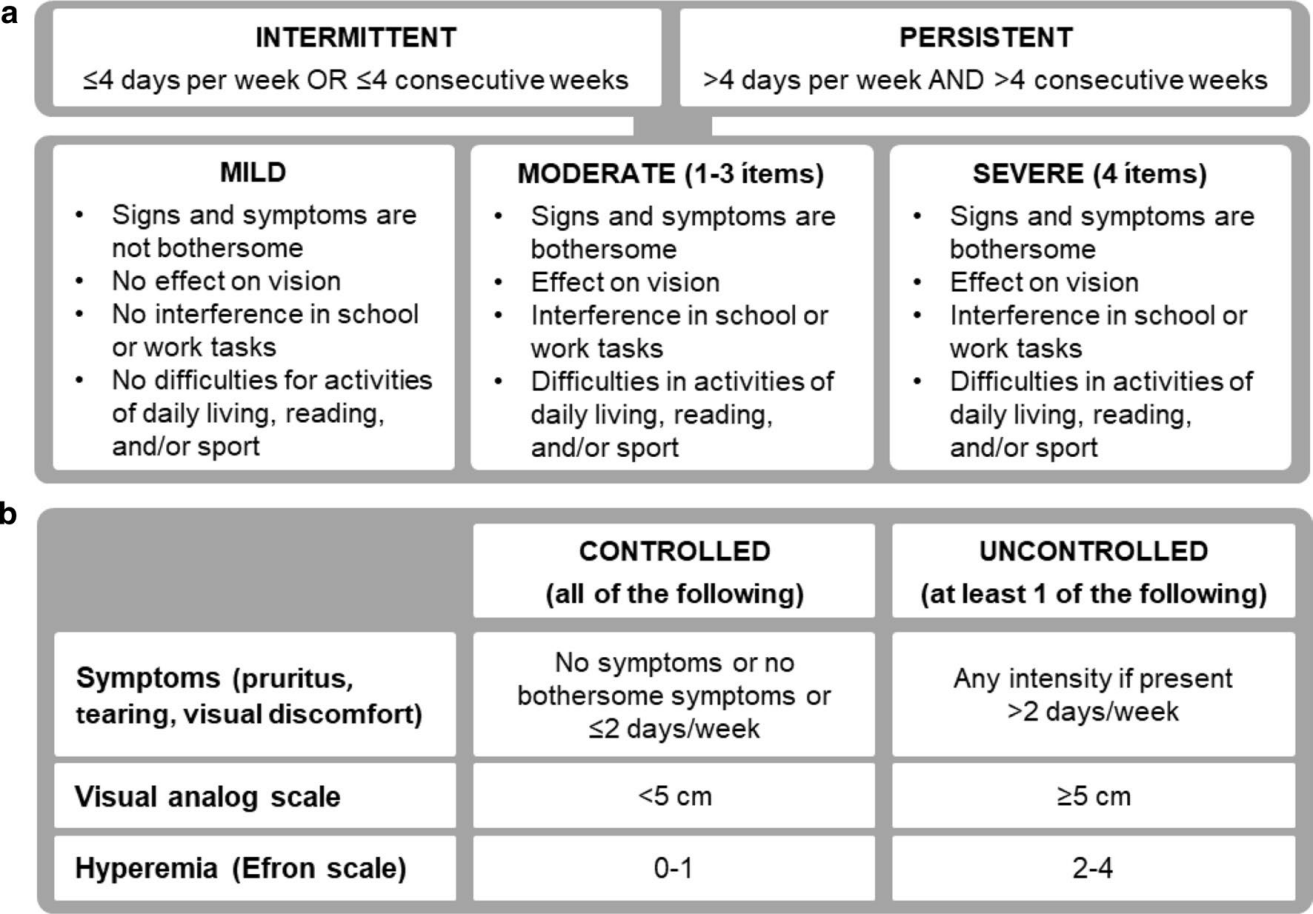
The criteria from the Spanish consensus document on allergic conjunctivitis (DECA). **a** Criteria for severity; **b** Criteria for control

In 2015, experts from the Spanish Societies of Allergology and Ophthalmology proposed a consensus of basic criteria that could be useful for both specialists and primary care physicians to facilitate the diagnosis, classification, control and treatment of patients with AC [13]. This consensus (henceforth named DECA, for its acronym in Spanish) was based on the modified ARIA (mARIA) classification of AR [14] adapted to AC (Fig. 1). The mARIA criteria classified patients as mild (when no items are affected), moderate (involvement of 1, 2 or 3 items) and severe (involvement of the 4 items) in both untreated and treated patients [14, 15]. Accordingly, in the DECA classification three levels of severity were proposed (mild, moderate, severe) and the criteria for frequency were retained (intermittent/persistent).

The novel DECA criteria for control were based on the analysis of control criteria proposed for various allergic diseases [13]. AC was classified as controlled or uncontrolled based on 3 evaluation criteria: occurrence and frequency of ocular symptoms, a VAS score, and the degree of conjunctiva hyperemia as determined by the Efron scale [16].

The objective of this study was to validate the DECA criteria for classifying severity and control in AC patients, following the recommendations from the Consensus-based Standards for the Selection of Health Measurement Instruments (COSMIN) initiative [17], in a sample of AC patients included in an observational study on the control of allergic rhinitis in Spain (CORINA) study.

## Methods

This study was part of a larger observational, prospective, and multicenter real-life study to assess the epidemiology of patients with moderate and severe AR in Spain in terms of control (CORINA), carried out between November 2015 and October 2016. Research was performed by allergists and/or otorhinolaryngologists working at reference hospitals and health centers throughout Spain. To avoid a possible bias due to seasonality, each researcher included no more than 4 consecutive patients meeting the inclusion criteria each month until reaching the target figure of 15 patients per investigator. The study was non-interventional as the clinical decision to prescribe treatments for the management of AC was made according to the usual clinical practice and was prior and independent of any consideration of the possible participation of patients in this study. During the study, patients received standard medical care without analytical tests or other clinical procedures specific to the study. All included patients had to sign informed consent and be able to attend the follow-up visit. The study was approved by the Ethics Committee for Clinical Research of the Hospital Clínic de Barcelona.

### Patient population and study design

The inclusion criteria were age ≥ 18 years and a diagnosis of AC according to the criteria in the DECA document [13] (Fig. 1). All patients had moderate or severe AR according to mARIA criteria [14], with a reflective total nasal symptom score (rTNSS) of ≥ 8 (scale 0–12).

The patients made a first visit (baseline visit) and a second visit 4–5 weeks later (follow-up visit). We followed COSMIN recommendations, as this international consensus established how to measure and evaluate in a uniform and consensual way the different health instruments [17]. According to COSMIN, the DECA severity and control criteria were validated for:

1. Discriminant validity was evaluated by comparing differences in the reflective total ocular symptom score (rTOSS), the rTNSS, question 3 of the rhinitis control assessment test (RCAT), a visual analogue scale (VAS), hyperemia scale and question 4 from the ESPRINT-15 questionnaire between patients classified in the different categories of severity or control using DECA criteria. Also, the percentage of patients classified in each category of severity using mARIA criteria was assessed for each category of AC severity by the DECA criteria.

2. Test–retest reliability was evaluated by analyzing the score agreement (kappa coefficient) in the basal and follow-up visits in those patients reporting equal AC severity or control. These patients were defined as those who had < 1.2 changes in the VAS score or ≤ 1 point in ESPRINT-15 question 4.

3. Responsiveness was assessed comparing the magnitude of the change in the different PROs (rTOSS, VAS, the Efron scale, and ESPRINT-15 question 4 for severity and rTOSS, ESPRINT-15 question 4, and RCAT question 3 for control) for three options of change in severity and control: worse, equal and improved.

### Patient-reported outcomes and assessments

The values of all the study variables were extracted from the patients’ medical records and from the study questionnaires filled out during the visits. Data was collected on demography, concomitant diseases and medication use, etiology of allergic sensitization, and severity and control of AC.

AC symptoms evaluated were pruritus, tearing and redness. Each of the symptoms was evaluated on a scale between 0 (no symptom) and 3 (severe symptom) and on a VAS (0–10 cm; 0 = no symptoms, 10 = maximum severity). Conjunctive hyperemia was measured with the grading scale by Efron (0–4; 0 = normal, 4 = severe) [16]. The rTOSS was calculated as the sum to the individual score at 3 symptoms, with a maximum score of 9. Question 3 of the Spanish-validated RCAT was used to evaluate control: “During the past week, how often did you have watery eyes?” (5–1; 5 = never, 1 = extremely often) [18, 19]. Question 4 of the Spanish version of the ESPRINT-15 questionnaire was used to evaluate affectation of quality of life: “In the past 2 weeks, how much have you been bothered by itchy eyes or having to rub your eyes?” (0–6; 0 = not at all; 6 = extremely) [20, 21].

AC severity was classified according to the DECA criteria based on the impact on 4 items: bothersome symptoms, affectation of vision, interference with academic or work tasks, or interference with daily activities, reading and/or sport (Fig. 1a) [13]. Based on these criteria, patients were classified as mild (no items affected), moderate (involvement of 1, 2 or 3 items) and severe (involvement of the 4 items). AC was also classified according to its duration in intermittent (up to 4 days/week or up to 4 consecutive weeks) or persistent (more than 4 days/week and more than 4 consecutive weeks).

The degree of AC control was assessed, according to the DECA criteria, by symptoms, VAS and the Efron scale for conjunctival hyperemia (Fig. 1b) [13]. AC was considered controlled if there were no symptoms or they were not bothersome 2 or more days per week, VAS < 5 cm and hyperemia scale was 0–1. It was considered uncontrolled if at least one of the following items was observed: symptoms with any intensity 2 or more days per week, VAS ≥ 5 cm and/or hyperemia scale was 2–4.

### Statistical methods

For the statistical analysis, the package SAS version 9.2 for Windows was used. The mean, median, standard deviation, minimum and maximum, 25th and 75th percentiles, and the number of valid cases were used for the description of continuous variables. The number and percentage of patients per response category was used for categorical variables. Prior to performing parametric tests, we applied statistical techniques to ensure compliance with the assumptions. In case the established assumptions were not met, non-parametric tests were employed. A level of statistical significance (p-value) of < 0.05 was used for all statistical tests.

## Results

The CORINA study recruited 252 patients [18]. Of these, 198 (78.6%) presented with AC symptoms in the basal visit, and 128 patients (50.8%) in the follow-up visit. The validation of the DECA criteria for severity and control was performed on this sample of 128 patients with AC symptoms and who attended both the baseline and the follow-up visits. Table 1 shows the baseline characteristics of the patients included in this study. The mean age of the AC patients was 34.4 ± 12.1 years and 72.7% were women. The mean time elapsed between the date of diagnosis and the study was 6.3 ± 9.7 years. At the basal visit, 94.5% of patients presented pruritus, 85.9% tearing, and 85.9% redness. All patients presented moderate or severe AR (100%) and 41.4% had concomitant asthma, 10.9% atopic dermatitis, and 3.1% food allergy. The most frequent allergen sensitizations were to dust mites (57.7%) and grass pollens (45.5%), while 63% of the patients presented polysensitization. Regarding type of AC sensitization, for 25.2% of the patients it was seasonal, for 32.5% perennial, and for 42.3% both. Table 1 shows the treatment followed by the patients at baseline, which the specialist adjusted according to current guidelines at baseline, modifying it in 63.3% of cases and initiating it in 20.3%.

**Table 1:** Baseline characteristics of the patients included in the DECA criteria for AC severity and control validation study (N = 128)

Age, years, mean (SD)	34.4 (12.1)
Gender, female, N (%)	93 (72.7)
Allergic comorbidities, N (%)	
AR	128 (100.0)
Asthma	53 (41.4)
Atopic dermatitis	14 (10.9)
Urticaria	11 (8.6)
Contact dermatitis	4 (3.1)
Food allergy	4 (3.1)
Other	14 (10.9)
Type of AC sensitization, N (%)	
Perennial	40 (32.5)
Seasonal	31 (25.2)
Both	52 (42.3)
rTOSS score, mean (SD)	5.4 (2.3)
Hyperemia (Efron scale), N (%)	
Normal	26 (20.3)
Trace	62 (48.4)
Mild	29 (22.7)
Moderate	11 (8.6)
AC duration, N (%)	
Intermittent	44 (34.4)
Persistent	84 (65.6)
Items reported (DECA), N (%)	
Bothersome symptoms	116 (90.6)
Affecting vision	39 (30.5)
Interference with academic or work tasks	54 (42.2)
Interference with daily activity, reading, sport	66 (51.6)
Severity (DECA criteria), N (%)	
Mild (no items affected)	10 (7.8)
Moderate (1–3 items affected)	93 (72.7)
Severe (4 items affected)	25 (19.5)
Control (DECA criteria), N (%)	
Controlled	27 (21.1)
Not controlled	101 (78.9)
Ophthalmic treatments at baseline, N (%)	
Azelastine	34 (26.6)
Ketotifen	11 (8.6)
Olopatadine	3 (2.3)
Levocabastine	1 (0.8)
Others	1 (0.8)
Nasal treatments at baseline, N (%)	
Budesonide	2 (1.6)
Fluticasone	12 (9.4)
Mometasone	22 (17.2)
Triamcinolone	1 (0.8)
Fluticasone furoate	43 (33.6)
Fluticasone/azelastine combination	41 (32.0)

AC: allergic conjunctivitis; AR: allergic rhinitis; DECA: Spanish document on allergic conjunctivitis; rTOSS: reflective total ocular symptom score; SD: standard deviation

### Validation of the DECA criteria for AC severity

#### Discriminant validity

**Fig. 2:**
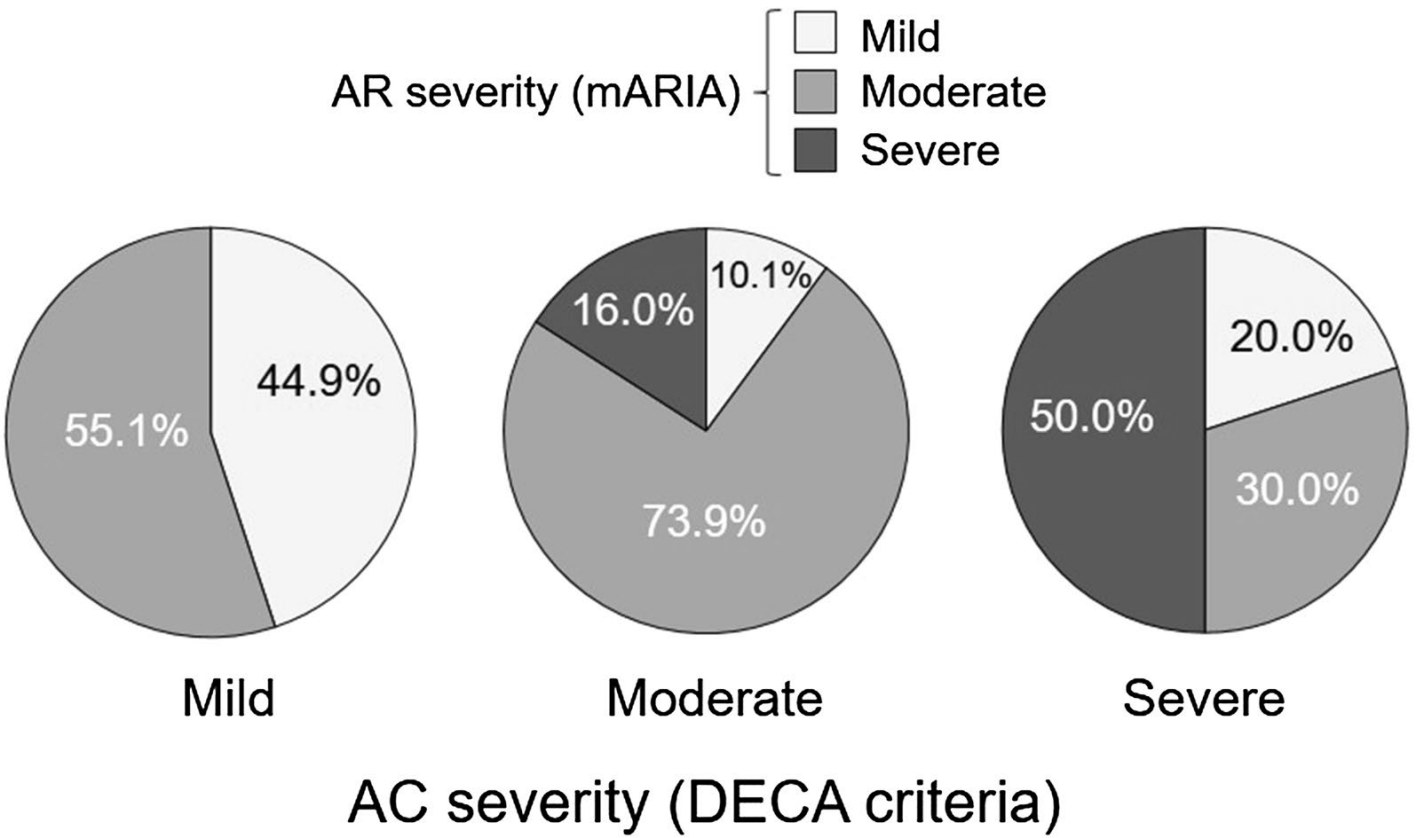
Percentage of patients in each allergic conjunctivitis (AC) severity category using DECA criteria (mild, moderate, or severe) and for each allergic rhinitis (AR) severity category by mARIA

Table 2 shows the scores of the different validated PROs when assessed according to the severity categories defined by using the DECA criteria. A highly significant discriminant validity of the DECA criteria for severity was found when comparing these scores. The percentage of patients classify similarly regarding severity with the mARIA criteria and the DECA criteria also showed a good balance facing both systems (Fig. 2).

**Table 2:** Mean (standard deviation) for the scores of the different validated patient reported outcomes assessed in the severity categories classified by using the DECA criteria

	DECA criteria			p
	Mild	Moderate	Severe	
Ocular symptoms (0–3), mean (SD)				
Pruritus	0.77 (0.83)	1.61 (0.84)	1.89 (1.05)	< 0.0001
Tearing	0.47 (0.78)	1.25 (0.89)	1.78 (1.09)	< 0.0001
Redness	0.43 (0.68)	1.28 (1.01)	1.78 (0.83)	< 0.0001
rTOSS score (0–9), mean (SD)	1.66 (1.98)	4.17 (2.28)	5.44 (2.65)	< 0.0001
Ocular symptoms by VAS (0–10 cm), mean (SD)				
Pruritus	1.70 (2.17)	4.48 (2.94)	5.30 (2.86)	< 0.0001
Tearing	1.45 (2.30)	3.80 (2.95)	4.79 (3.20)	< 0.0001
Redness	1.35 (2.41)	3.94 (3.05)	4.56 (3.03)	< 0.0001
VAS global score	1.82 (2.21)	4.87 (2.88)	6.14 (3.01)	< 0.0001
Efron hyperemia scale (0–4), mean (SD)	0.10 (0.31)	0.93 (0.81)	1.20 (1.14)	< 0.0001
ESPRINT-15 (0–6), mean (SD) Question 4*	1.39 (1.51)	3.32 (1.71)	4.10 (1.37)	< 0.0001

DECA: Spanish document on allergic conjunctivitis; ESPRINT-15: Spanish allergic rhinitis quality of life questionnaire; rTOSS: reflective total ocular symptom score; VAS: visual analogue scale; SD: standard deviation

*ESPRINT-15 Question 4: “In the past 2 weeks, how much have you been bothered by itchy eyes or having to rub your eyes?”

#### Test–retest reliability

Thirty-three patients had the same AC severity as assessed by the VAS, resulting in a kappa value of 0.45 (72.7% agreement) with the DECA severity criteria, and 56 patients for the ESPRINT-15 question 4, kappa value of 0.33 (62.5% agreement), reflecting a fair test–retest reliability.

#### Responsiveness

**Fig. 3:**
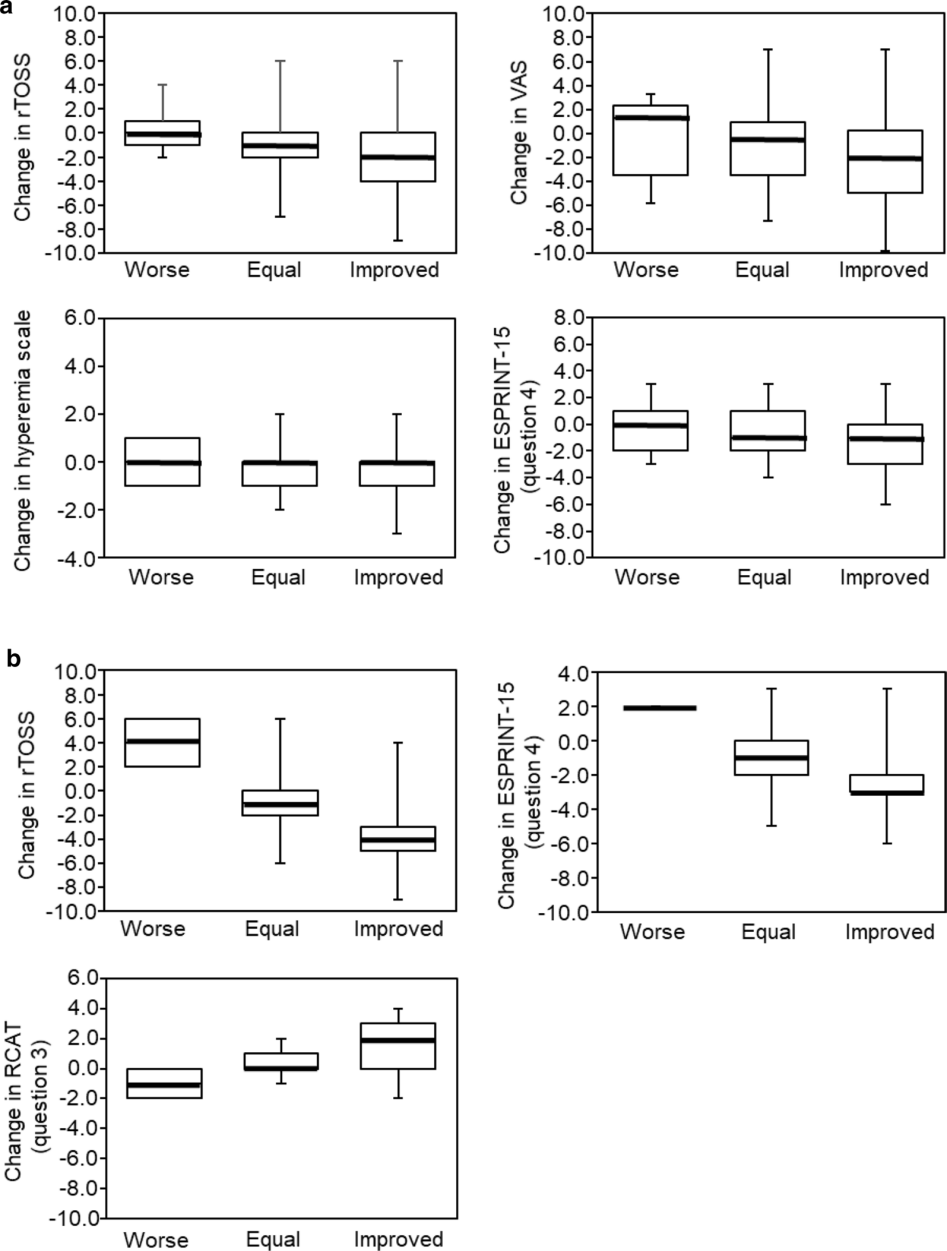
Boxplots showing the DECA criteria score changes (median, 25th and 75th interquartile values) between visits for AC patients, which were categorized as worse, equal, or improved compared to baseline. **a** Comparisons for PROs evaluating severity. The differences are statistically significant for rTOSS (p < 0.0001), VAS (p < 0.0001), hyperemia scale (p < 0.0002), and ESPRINT-15 question 4 (p < 0.0001). **b** Comparisons for PROs evaluating control. The differences were statistically significant for rTOSS (p < 0.0001), ESPRINT-15 question 4 (p < 0.0001), and RCAT question 3 (p < 0.0002)

The magnitudes of the change in the PROs scores for patients who worsened, improved or stayed the same in their AC severity were significantly different regarding rTOSS, VAS, the Efron hyperemia scale, and ESPRINT-15 question 4 (Fig. 3a), suggesting that the DECA criteria for severity showed good responsiveness to detect changes on symptoms, ocular signs and quality of life.

### Validation of the DECA criteria for AC control

Patients classified as controlled by the DECA criteria showed PROs scores significantly different from patients classified as uncontrolled, indicating a high discriminant validity of the DECA criteria for AC control (Table 3).

**Table 3:** Mean (standard deviation) of the different validated patient reported outcomes for controlled versus not controlled AC control assessed by the DECA criteria

	DECA criteria		p
	Controlled	Not controlled	
Ocular symptoms (0–3), mean (SD)			
Pruritus	0.94 (0.86)	2.00 (0.71)	< 0.0001
Tearing	0.65 (0.89)	1.62 (0.82)	< 0.0001
Redness	0.60 (0.81)	1.76 (0.99)	< 0.0001
rTOSS score (0–9), mean (SD)	2.19 (2.25)	5.38 (1.97)	< 0.0001
Ocular symptoms by VAS (0–10 cm), mean (SD)			
Pruritus	0.94 (0.86)	2.00 (0.71)	< 0.0001
Tearing	0.65 (0.89)	1.62 (0.82)	< 0.0001
Redness	0.60 (0.81)	1.76 (0.99)	< 0.0001
ESPRINT-15 (0–6), mean (SD) Question 4*	1.75 (1.60)	4.33 (1.40)	< 0.0001
RCAT (5-1), mean (SD) Question 3**	3.68 (1.13)	2.20 (0.96)	< 0.0001

DECA: Spanish document on allergic conjunctivitis; ESPRINT-15: Spanish allergic rhinitis quality of life questionnaire; RCAT: rhinitis control assessment test; rTOSS: reflective total ocular symptom score; VAS: visual analogue scale

* ESPRINT-15 Question 4: “In the past 2 weeks, how much have you been bothered by itchy eyes or having to rub your eyes?”

** RCAT Question 3: “During the past week, how often did you have watery eyes?”

Patients who did not report changes in AC control, when assessed by DECA criteria, between the basal and follow-up visits (N = 15) showed a similar score in PROs (Kappa value of 0.59; 80% agreement), proving a good test–retest validity.

The changes in PROs scores for patients who changed their level of control assessed by DECA criteria were significantly and relevantly higher than patients who do not, showing a good responsiveness (Fig. 3b).

## Discussion

This study successfully validated the DECA criteria for severity and control of AC according to the requirements established in the COSMIN: discriminant validity, test–retest reliability, and responsiveness [17]. The data obtained in the CORINA study described here suggest that the DECA criteria could be used to rapidly assess AC severity and control of the disease in patients with AC symptoms.

Despite the increase in allergic eye diseases in recent decades and the socioeconomic impact generated by AC [3–5, 22], it is commonly accepted that AC is often underdiagnosed and undertreated [6]. This could be due in part to the highly frequent comorbidity of AC with other more severe allergic diseases such as asthma [23]. In our study we also observed this frequent comorbidity, as asthma was present in 41.4% of the patients and atopic dermatitis in 10.9% (in addition to rhinitis which, for methodological reasons, was present in all patients). Another possible reason for the underdiagnosis of AC could be the lack of an unanimously agreed classification criteria. Additionally, in most epidemiological studies on allergic diseases the eye and nasal symptoms have been treated as a single clinical entity [24]. For these reasons, in an effort to bring together the classification criteria of AC and AR and to unify nomenclature on AC, the DECA consensus document proposed a new classification of AC and also added a proposal for the definition of AC control [13].

In this study some relevant properties of the DECA criteria were assessed. The discriminant validity for disease severity, as shown in Table 2, reflected a high capacity to differentiate mild, moderate, and severe patients. The extent by which AC is associated with AR could also be observed when patient classification by the DECA criteria were compared with the mARIA criteria. Although AR severity and AC severity do not necessarily have to follow parallel classifications, in our study we observe a good agreement between the two, suggesting that these instruments are effective and can simplify and expedite the classification of both diseases [25]. Additionally, we find it is highly useful to differentiate AC severity in three categories (mild, moderate, and severe), as differentiating moderate to severe patients can reduce the heterogeneity in the higher severity status [14, 15].

The capacity of the DECA criteria to discriminate patients according to the degree of AC control can be observed in Table 3, clearly suggesting their validity. Longitudinal validity and responsiveness for the DECA criteria severity and control were also acceptable and suggest that they could be useful in the evaluation and follow-up of treated patients.

This validation study had some limitations. First, it only included patients with a diagnosis of moderate to severe AR or that patients with ocular symptoms alone were not included. Second, a methodological limitation derives from the lack of a standard and normalized classification for eye symptoms related to AC. For this reason, in our validation we could only compare the DECA criteria with other validated instruments previously applied to various aspects of symptom severity and quality of life formally designed for AR. We have also used the specific ocular items of the ESPRINT-15 and RCAT questionnaires (questions 4 and 3, respectively), but these items were not designed to be used in isolation and therefore their reliability has not been validated. Similarly, comparison with other classification schemes previously proposed before has not been possible, as they have not been validated.

One of the strengths of this study is that it showed for the first time the validation of an instrument specific for AC control, following the COSMIN recommendations for reliability, validity and responsiveness [17]. It has been suggested that measurements of disease control should be reproducible and easily implementable in everyday practice and focused on the disease’s impact in daily life [9]. In this regard, the DECA criteria are designed to be a practical tool used by primary health practitioners and specialists alike. Further, the proposed clinical classification of AC severity is consistent and complementary with that currently in use for AR severity. The unification of criteria for the evaluation of severity and control is important in the development of diagnostic and treatment guidelines for common use by primary care physicians, allergists, and ophthalmologists, as a multidisciplinary communication is necessary for the optimal management of AC patients [1, 6, 9]. In this regard, the validated instruments described here could also be used to rapidly screen these patients with AC control problems and could help patients to communicate with health care practitioners about their disease and their response to treatment.

## Conclusion

This study validated with good results the criteria for severity and control assessment proposed in DECA, making this a potentially useful tool for physicians and patients in the evaluation of eye symptoms and follow-up of therapies.

## Data Availability

Data is available from the corresponding author upon reasonable request.

## Abbreviations

AC: Allergic conjunctivitis
AR: Allergic rhinitis
ARIA: Allergic rhinitis and its impact on asthma
COSMIN: Consensus-based standards for the selection of health measurement instruments
DECA: Consensus document on allergic conjunctivitis (Spanish acronym for “Documento de consenso sobre conjuntivitis alérgica”)
ESPRINT-15: Spanish allergic rhinitis quality of life questionnaire
mARIA: Modified allergic rhinitis and its impact on asthma
PRO: Patient-reported outcome
QoL: Quality of life
RCAT: Rhinitis control assessment test
rTNSS: Reflective total nasal symptom score
rTOSS: Reflective total ocular symptom score
VAS: Visual analogic scale
